# Supplementary material for: Detection of Single Molecules Using Stochastic Resonance of Bistable Oligomers
Source: Nanomaterials (Basel). 2020 Dec 15;10(12):2519. doi: 10.3390/nano10122519 (PMC7765484; doi:10.3390/nano10122519)
Supplement: Supplementary file 1 [file nanomaterials-10-02519-s001.pdf]

nanomaterials

Supplementary Materials

Detection of Single Molecules Using Stochastic Resonance of Bistable Oligomers

Anastasia Markina *, Alexander Muratov, Vladislav Petrovskii and Vladik Avetisov *

N. N. Semenov Federal Research Center for Chemical Physics Russian Academy of Sciences, Kosygina 4, 119991 Moscow, Russia; ad.muratov@physics.msu.ru (A.M.); petrovskyy@gmail.com (V.P.)

* Correspondence: markina@mpip-mainz.mpg.de (A.M.); avetiov@chph.ras.ru (V.A.)

S1. Simulation protocol

Morphology simulations were performed using the GROMACS simulation package. Details of the parameters used for non-bonded interactions are presented in Figure S1a. Long-range electrostatic interactions were treated using a smooth particle mesh Ewald technique. All calculations were performed in the NVT ensemble using the canonical velocity-rescaling thermostat, as implemented in the GROMACS simulation package.

A random initial configuration was used to start the simulation. To reach an equilibrated morphology, the simulation was initialized using 16,742 water molecules and one NIPMAm oligomer in a syndiotactic configuration 30 monomeric units long. These molecules were modeled inside a box measuring 5.0 × 3.0 × 3.0 nm first exposed to a thermal bath at 290 K for 50 ns with a simulated time step of 0.001 ps (n = 5 independent trajectories). The simulation was repeated in a larger box (8.0 × 8.0 × 8.0 nm), confirming that results were not influenced by box size. An image of the larger simulation box (8.0 × 8.0 × 8.0 nm) is shown in Figure S1b.

To study how the oligomers respond to a power load, system simulation was continued for an additional 150 ns (n = 2 independent trajectories). Model error was estimated using the full width at 50% of the distribution curve maximum. Various water models (SPCE, TIP3P, TIP4P) were used, showing that results are independent of the model applied. Significant differences in the spontaneous oscillations of the end-to-end distance R_e under the critical load ($F = 400\,pN$ Figure S1c) were not found.

a.

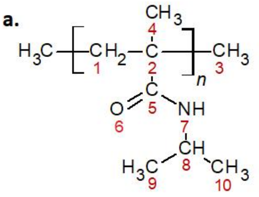

№	FF	q
1	opls_136	-0.12
2	opls_139	0
3, 4, 9, 10	opls_135	-0.18
5	opls_235	0.5
6	opls_236	-0.5
7	opls_238	-0.5
8	opls_229	0.14
H7	opls_241	0.3
H	opls_140	0.06

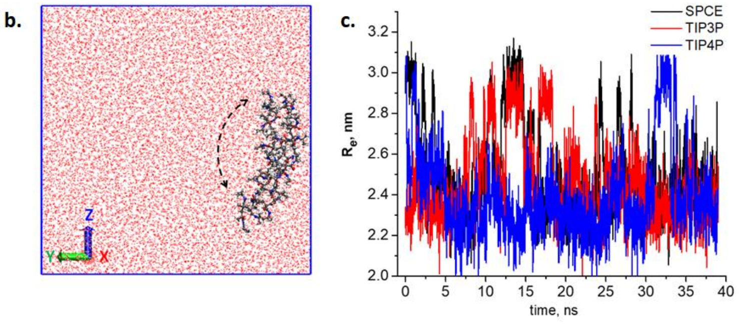

Figure S1. Simulation details. a) Non-bonded interaction parameters from the OPLS-AA force field used in the simulation; b) the YZ-plane of the simulation box (8.0 × 8.0 × 8.0 nm; 50,888 particles); and c) oscillations of the end-to-end distance R_e for various water models (SPCE, TIP3P, and TIP4P).

The simulation workflow consisted of the following:

1) An "open" conformation of oligo-30s-NIPMAm was obtained by equilibrating the oligomer at 290 K. This configuration of oligo-30s-NIPMAm was oriented in the YZ-plane and fixed in the X dimension.

2) The center of mass for the first monomeric unit in the oligomeric chain was fixed using a spring potential of $k = 100kJ / molnm^2$. No other specific constraints for bond length or atom position were applied.

3) The longitudinal (compressing) load *F* was applied to the center of mass of the last monomer unit and directed toward the attraction point, located at the center of mass of the first monomeric unit along the vector connecting the left and right ends of the molecule. Note that the orientation of this vector changed over time because the first monomeric unit was fixed while the 30th monomeric unit was mobile.

4) To study stochastic resonance, an oscillating force was realized by setting a charge (+1) at one end of the oligomer and a compensative charge (–1) at the other end. An external oscillating electrical field $E = E_0 \cos \omega t$ was directed along the compressive force F. The period of the harmonic electrical field was close to the period of random fluctuation $T = 5ns$, and the amplitude was $E_0 = 0.2V / nm$.

5) The lateral load G was applied to the center of mass of the 16th monomer unit (the middle part of the oligo-NIPMAm). Hysteresis (Figure 4a) was observed when a lateral load was added to the system under critical compression. In this case, the vibration region (stochastic resonance) depended on increasing or decreasing the lateral load.

6) To represent stochastic resonance, the end-to-end distance under a compressing force F and a lateral force G that were fixed near their critical values was plotted against time.

S2. Sensing regime

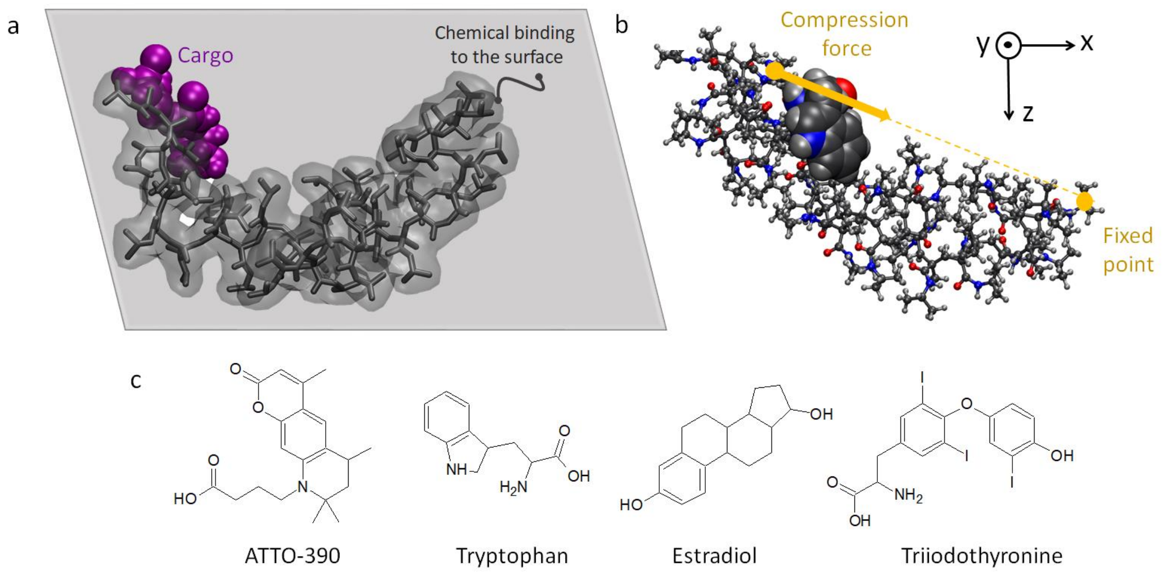

Figure S2. Model of the sensing element. (**a**) Structure of oligo-NIPMAm, hydrophobic and hydrophilic blocks. (**b**) Computational model of oligo-30s-NIPMAm under an applied longitudinal F load. (**c**) Chemical structures of hydrophobic small molecules under study: the dye ATTO-390, the amino acid tryptophan, and the hormones estradiol and triiodothyronine.

To apply an additional load to the system, the following workflow was used:

1) An "open" conformation of oligo-30s-NIPMAm at a temperature of 290 K was oriented in the YZ-plane and fixed in the X dimension (See Figures S2a and b).

2) Small molecules (one, two, or three) were placed in the vicinity of the oligo-NIPMAm molecule. After ~10 ns of equilibration, the small molecule was absorbed on the oligo-NIPMAm (see Figure S3 for minimum distances for various types of molecules).

3) The longitudinal load applied to the oligomer was then bent as described in the simulation protocol. Alternatively, the oligomer can be placed in a closed state, equilibrated at 320 K, then loaded in opposite directions. Results were not qualitatively different in this scenario.

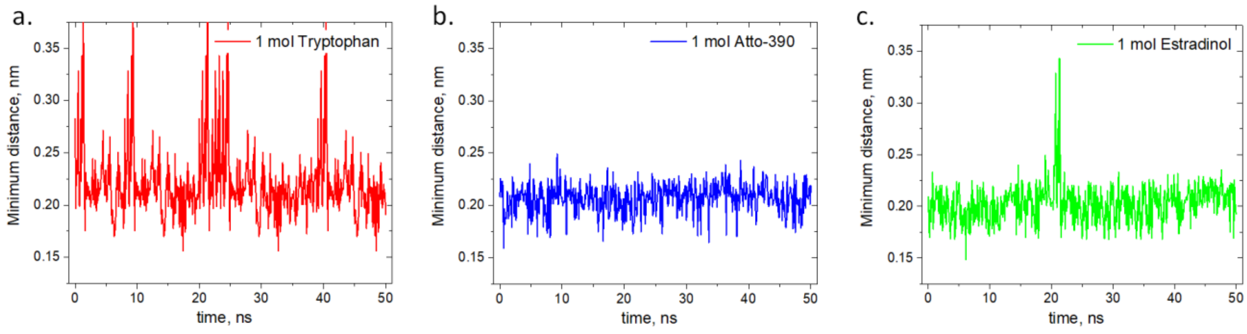

Figure S3. Adsorption of molecular cargo. The minimum distance is between any atom of oligo-NIPMA and any atom in the cargo molecule tryptophan (**a**), ATTO-390 (**b**), or estradiol (**c**).

The behaviors of oligo-NIPAM loaded with various types of cargos are shown in Figures S4-S6.

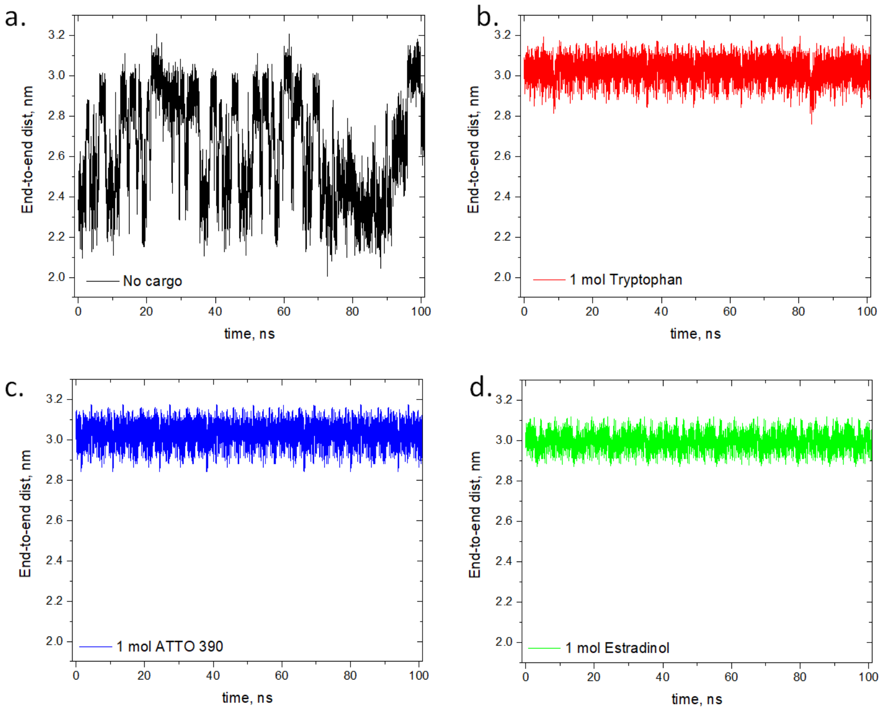

Figure S4. Sensing regime. Molecular dynamic trajectories were determined for the system under the critical compression $F = 375\,pN$ and without a cargo molecule (**a**) and with one molecule of tryptophan (**b**), ATTO-390 (**c**), or estradiol (**d**).

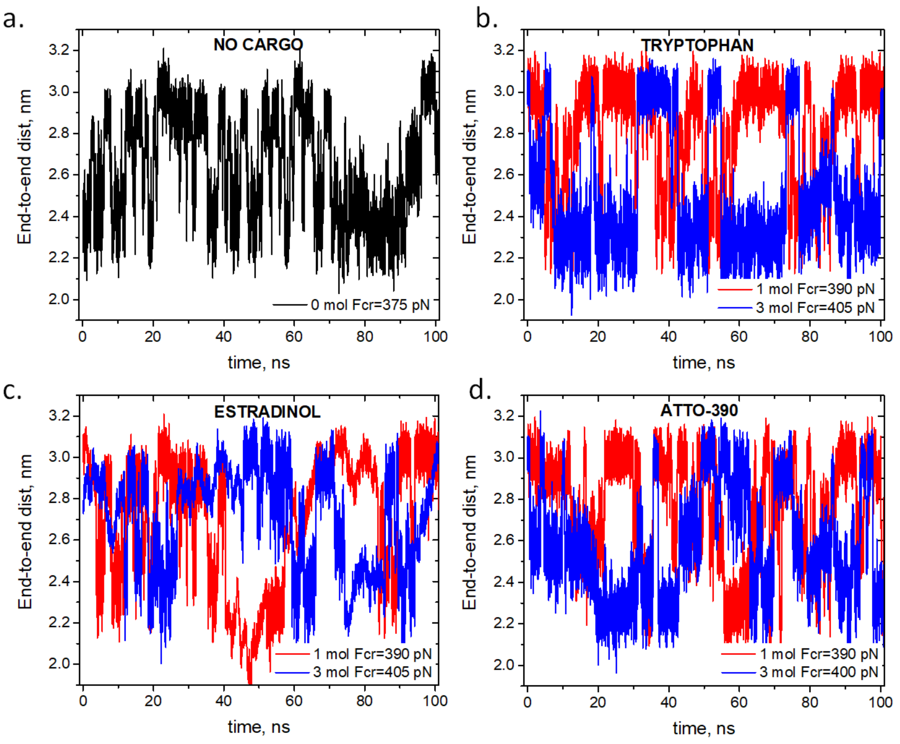

Figure S5. Molecular dynamic trajectories. The end-to-end distance of the system was determined for the system under the critical compression $F = 375\,pN$ and without a cargo molecule (a) and with one or three molecules of tryptophan (**b**), estradiol (**c**), or ATTO-390 (**d**).

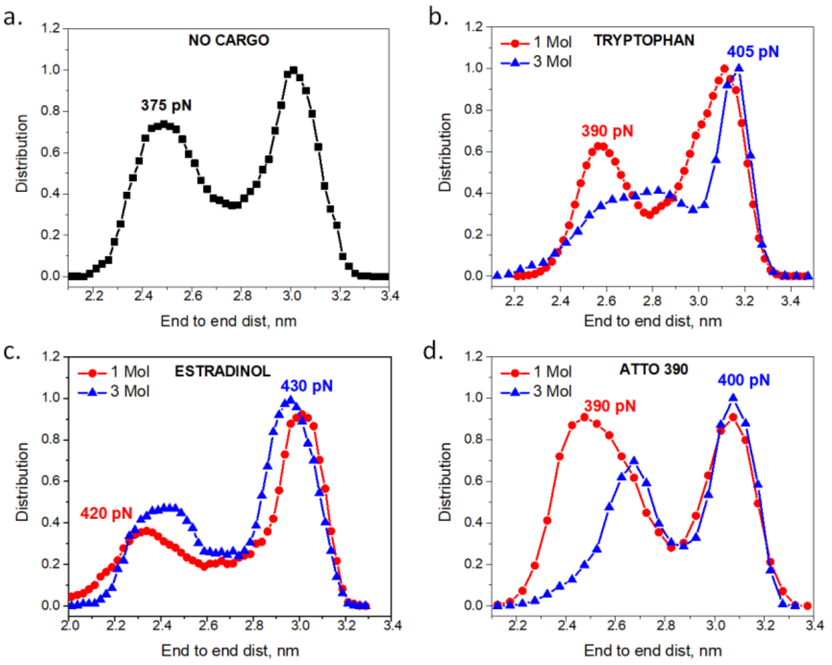

Figure S6. Spontaneous vibrations diagrams. The statistical weights of visits to the opened and closed states were determined under the critical compression $F = 375\,pN$ and without a cargo molecule (**a**) and with one or three molecules of tryptophan (**b**), estradiol (**c**), or ATTO-390 (**d**).

S3. Sensitivity

The sensing regime is sensitive to the mass of the binding small molecule and its binding affinity (see Figure S7). The number of sites and the hydrogen binding affinity differed between the small molecules. Binding energies were obtained using an umbrella sampling technique.

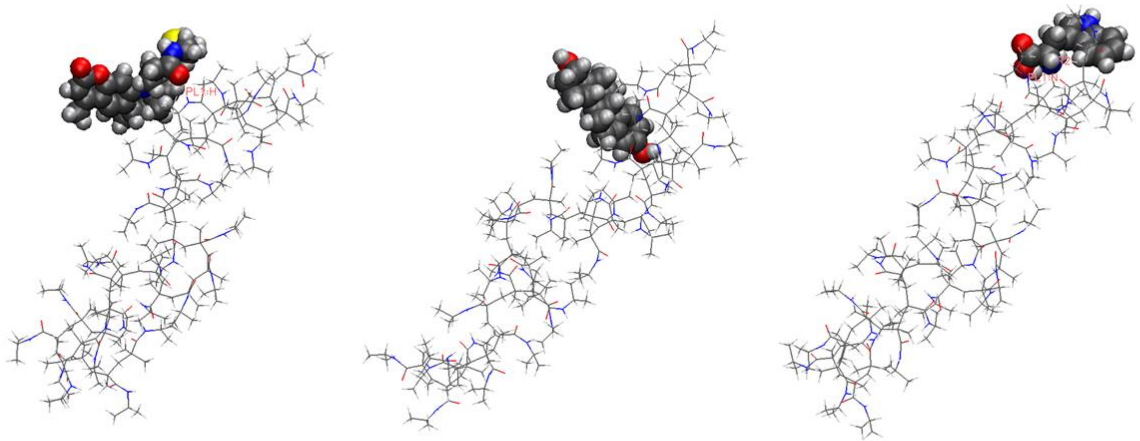

ATTO-390 ($M = 390\ g/mol$)	ESTRADINOL ($M = 272\ g/mol$)	TRYPTOPHAN ($M = 204\ g/mol$)
$F = 390\ pN$	$F = 420\ pN$	$F = 390\ pN$
$f = \frac{F}{M} = 1.13\ pN\ mol/g$	$f = \frac{F}{M} = 1.54\ pN\ mol/g$	$f = \frac{F}{M} = 1.94\ pN\ mol/g$
$E = 4\ kcal/mol$	$E = 7\ kcal/mol$	$E = 10\ kcal/mol$
1 hBond	1 hBond	2 hBond

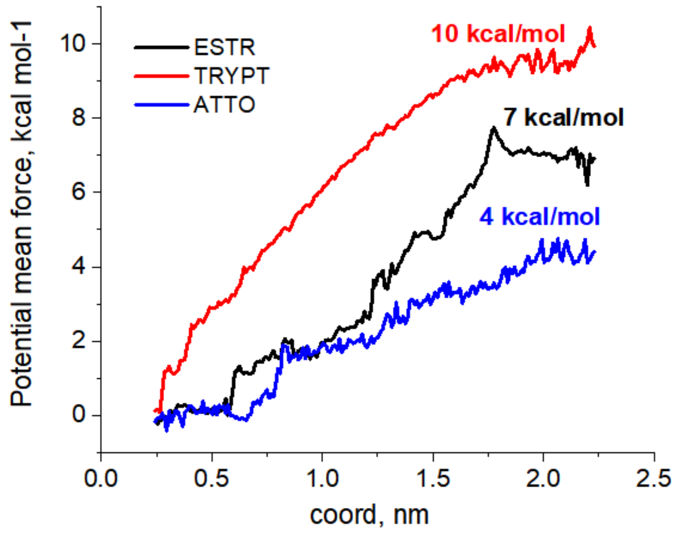

Figure S7. Binding energies and the number of hydrogen bonds in each molecule studied. The molar mass, critical force for spontaneous vibrations, critical force normalized to molar mass, binding energy, and number hydrogen bonds is shown for each molecule.
